# Cytokine-Neuroantigen Fusion Proteins as a New Class of Tolerogenic, Therapeutic Vaccines for Treatment of Inflammatory Demyelinating Disease in Rodent Models of Multiple Sclerosis

**DOI:** 10.3389/fimmu.2012.00255

**Published:** 2012-08-20

**Authors:** Mark D. Mannie, J. Lori Blanchfield, S. M. Touhidul Islam, Derek J. Abbott

**Affiliations:** ^1^Department of Microbiology and Immunology, East Carolina UniversityGreenville, NC, USA; ^2^Department of Microbiology and Immunology, Emory University, AtlantaGA, USA

**Keywords:** multiple sclerosis, experimental autoimmune encephalomyelitis, tolerogenic therapeutic vaccine, cytokine-neuroantigen fusion protein, immunological tolerance, interferon-beta, granulocyte-macrophage colony-stimulating factor, autoimmune demyelination

## Abstract

Myelin-specific induction of tolerance represents a promising means to modify the course of autoimmune inflammatory demyelinating diseases such as multiple sclerosis (MS). Our laboratory has focused on a novel preclinical strategy for the induction of tolerance to the major encephalitogenic epitopes of myelin that cause experimental autoimmune encephalomyelitis (EAE) in rats and mice. This novel approach is based on the use of cytokine-NAg (neuroantigen) fusion proteins comprised of the native cytokine fused either with or without a linker to a NAg domain. Several single-chain cytokine-NAg fusion proteins were tested including GMCSF-NAg, IFNbeta-NAg, NAgIL16, and IL2-NAg. These cytokine-NAg vaccines were tolerogenic, therapeutic vaccines that had tolerogenic activity when given as pre-treatments before encephalitogenic immunization and also were effective as therapeutic interventions during the effector phase of EAE. The rank order of inhibitory activity was as follows: GMCSF-NAg, IFNbeta-NAg > NAgIL16 > IL2-NAg > MCSF-NAg, IL4-NAg, IL-13-NAg, IL1RA-NAg, and NAg. Several cytokine-NAg fusion proteins exhibited antigen-targeting activity. High affinity binding of the cytokine domain to specific cytokine receptors on particular subsets of APC resulted in the concentrated uptake of the NAg domain by those APC which in turn facilitated the enhanced processing and presentation of the NAg domain on cell surface MHC class II glycoproteins. For most cytokine-NAg vaccines, the covalent linkage of the cytokine domain and NAg domain was required for inhibition of EAE, thereby indicating that antigenic targeting of the NAg domain to APC was also required *in vivo* for tolerogenic activity. Overall, these studies introduced a new concept of cytokine-NAg fusion proteins as a means to induce tolerance and to inhibit the effector phase of autoimmune disease. The approach has broad application for suppressive vaccination as a therapy for autoimmune diseases such as MS.

## Introduction: Immunological Tolerance to CNS Myelin as a Therapy for MS

Multiple sclerosis (MS) is an inflammatory demyelinating disease of the CNS (Nylander and Hafler, 2012). A defining hallmark of the disease is the formation of multiple discrete inflammatory lesions and focal demyelination in perivascular and periventricular sites of CNS white matter. These demyelinating lesions are marked by infiltration of activated mononuclear cells and are associated with the appearance of neurologic deficits. MS is also marked by significant involvement in CNS gray matter with axonal loss, cortical atrophy, and cognitive dysfunction (Calabrese et al., 2011). MS is considered to be an autoimmune disorder caused by T cells specific for immunodominant self-epitopes of myelin and other CNS antigens (Severson and Hafler, 2010). These autoreactive T cells are postulated to migrate across the blood-brain barrier into the CNS and undergo re-activation upon T cell antigen recognition of endogenous CNS epitopes. These activated T cells then secrete pro-inflammatory cytokines and chemokines to recruit inflammatory macrophages and other leukocytes from the blood to initiate focal demyelination and CNS dysfunction.

A central goal for the field of autoimmunity is to optimize strategies of antigen-specific tolerance induction as a therapy for chronic autoimmune disorders (Leech and Anderton, 2008; Sabatos-Peyton et al., 2010; Nepom et al., 2011). The goal is to derive antigen-specific vaccines to generate a suppressive immunological memory specific for particular target self-antigens. Realization of this goal will provide a potentially curative intervention that would reverse the pathological autoimmune response and thereby preempt the need for chronic administration of broad-spectrum immunosuppressive drugs. Thus, new tolerogenic strategies are needed to maximize reliability and efficiency of antigen-specific tolerogenic vaccines that may be amenable for use in humans.

Strategies of myelin-specific tolerance induction may thereby provide a means to develop more effective therapies for MS. Tolerogenic vaccine strategies would be disease-specific and would be based on the imposition of regulatory constraints on the dominant pathogenic clones responsible for MS. However, substantial hurdles exist. First and most importantly, the field currently lacks a valid and reliable means to induce myelin-specific tolerance in patients afflicted with MS. Second, myelin-specific tolerance regimens must not lead to inadvertent encephalitogenic sensitization, autoantibody formation, or anaphylactic sensitivity. Third, MS in different patients may be driven by autoreactivity against unique and perhaps non-overlapping sets of pathogenic myelin epitopes. Fourth, during the course of disease, “epitope-spreading” may generate an ever broadening polyclonal repertoire that targets an expanding multiplicity of myelin epitopes. Lastly, MS may transition from an immunological inflammatory disease amenable to immunological intervention to a neurodegenerative disease resistant to immunomodulatory approaches. Meaningful solutions to these challenges will stem from new technologies that profile the myelin-specific T cell specificities early during the course of disease coupled with new strategies to reliably induce tolerance to those myelin epitopes. Thus, an important part of this strategy will be to engineer immunosuppressive vaccines based on a robust platform that can induce reliable and potent tolerance in both non-inflammatory and overtly inflammatory environments. Despite the hurdles, myelin-specific induction of immunological tolerance represents the most promising path to specifically modify the course of autoimmune inflammatory demyelinating disease and thereby circumvent the need for broad-spectrum immunosuppression. Myelin-specific tolerance regimens promise qualitative improvements in clinical efficacy, therapeutic longevity, and cost-effectiveness without the adverse consequences of a compromised immune system.

Experimental autoimmune encephalomyelitis (EAE) represents a widely used preclinical model to test tolerogenic vaccines as candidates for potential translation for treatment of MS (Wekerle, 2008). EAE represents a valid model of the major pathophysiologic and regulatory mechanisms underlying anti-myelin autoimmunity in mammals. EAE can be induced by active immunization with dominant encephalitogenic epitopes of myelin together with immunological adjuvants. Alternatively, EAE can be induced by adoptive transfer of activated encephalitogenic T cells. Overall, the disease is regulated by many of the same counter-regulatory molecules (e.g., IL-10, TGF-beta, CTLA-4, PD-1) and cellular regulatory circuits believed to be important in MS. Hence, EAE is useful as a testing ground for new experimental vaccines designed to drive the inhibitory circuits underlying active, infectious tolerance.

Three rodent models of EAE were used in our studies, with each model representing a qualitatively different disease course and a potentially different underlying immunoregulatory mechanism. Initial studies were performed in Lewis rats which recognize the 73–87 sequence of myelin basic protein (MBP) as the dominant encephalitogenic epitope. Lewis rats immunized with the MBP73–87 peptide exhibit an acute monophasic form of EAE which manifests as an acute ascending paralysis followed by a spontaneous, complete remission. In some cases, Lewis rats having an intense initial course of EAE may exhibit a secondary relapse but this relapse is characterized by very mild paralytic signs. In contrast, the C57BL/6 and SJL mouse models of EAE represent models of chronic disease. C57BL/6 mice, after immunization with the myelin oligodendrocyte glycoprotein (MOG)35–55 epitope and separate injections of the Pertussis toxin adjuvant, exhibit a chronic course of severe non-resolving paralysis. The lack of spontaneous recovery in C57BL/6 mice may reflect inefficient or compromised regulatory responses. SJL mice immunized with the proteolipid protein (PLP)139–151 epitope exhibit a chronic relapsing-remitting course of EAE. These mice exhibit a severe monophasic episode of EAE followed by a spontaneous recovery and a subsequent asynchronous series of relapses and spontaneous recoveries. Thus, these cytokine-NAg vaccines were studied in preclinical models of EAE representing monophasic, chronic-progressive, and relapsing-remitting courses in rat and mouse species.

## Cytokine-NAg Fusion Proteins as Tolerogenic, Therapeutic Vaccines

**Figure 1:**
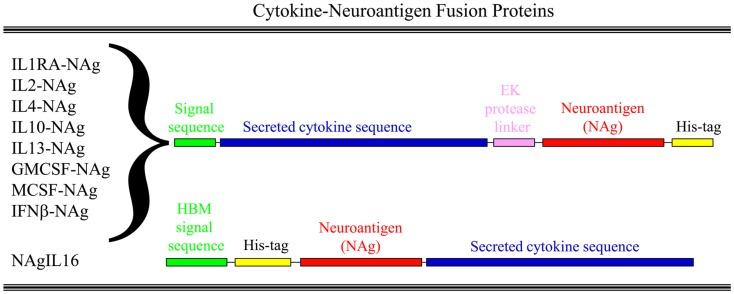
**Structure of cytokine-NAg vaccines**. Cytokine-NAg vaccine structure is portrayed including those incorporating native IL-1RA (IL-1 receptor antagonist), IL-2, IL-4, IL-10, IL-13, GM-CSF, M-CSF, or IFN-beta as the N-terminus or IL-16 as the C-terminus. Some fusion proteins, as discussed in the Table 1 legend, contained an enterokinase (EK) linker sequence.

Our laboratory has focused on a novel strategy for the induction of tolerance to the major encephalitogenic epitopes of myelin in both mouse and rat models of EAE (Mannie and Abbott, 2007; Mannie et al., 2007, 2009b; Blanchfield and Mannie, 2010; Abbott et al., 2011). The tolerogenic strategy is based on derivation of novel cytokine-NAg fusion proteins comprised of a native cytokine as the N-terminal domain fused either with or without a linker to a C-terminal NAg domain. The structural features of the single-chain cytokine-NAg fusion proteins are portrayed in Figure 1 and Table 1. The most effective cytokine domains for induction of tolerance were GM-CSF (Blanchfield and Mannie, 2010; Abbott et al., 2011), IFN-beta (Mannie et al., 2009b), IL-16 (Mannie and Abbott, 2007), and IL-2 (Mannie et al., 2007). In all cases, the cytokine domains were syngeneic with the species of the EAE model. The NAg domain contained the dominant epitope of the myelin protein responsible for induction of EAE in the given species and strain of rodent. In one case (NAgIL16), the NAg and cytokine domains were switched as the N-terminus and C-terminus respectively to preserve optimal activity of the cytokine. The cytokine domains of these vaccines had essentially the full activity of the free cytokine, and the NAg domain was efficiently processed and presented on MHC class II (MHCII) glycoproteins to NAg-specific T cells. Thus, the covalent cytokine-NAg linkage did not interfere with the independent activities of either the cytokine or NAg domain.

**Table 1:** **Selected cytokine-NAg vaccines tested for induction of tolerance in EAE**.

Cytokine-NAg vaccine	Species of cytokine	EAE model	NAg domain^a^	Optimized Kozak site^b^	Signal sequence^b^	Linker^c^	Expression system
GMCSF-NAg	Rat	Lewis rat	MBP69–87	No	mouse GM-CSF	Not included	Baculovirus
GMCSF-NAg	Mouse	SJL	PLP139–151	Yes	Native	Not included	Human
GMCSF-NAg	Mouse	C57BL/6	MOG35–55	Yes	Native	Not included	Human
IFNbeta-NAg	Rat	Lewis rat	MBP69–87	Yes	Native	EK linker essential	Human
IFNbeta-NAg	Mouse	SJL	PLP139–151	Yes	Native	EK linker essential	Human
NAgIL16	Rat	Lewis rat	MBP69–87	No	HBM	Not included	Baculovirus
MCSF-NAg	Rat	Lewis rat	MBP69–87	No	Native	Not included	Baculovirus
IL2-NAg	Rat	Lewis rat	MBP73–87	Yes	Native	Not needed	Baculovirus
IL4-NAg	Rat	Lewis rat	MBP73–87	No	Native	Not needed	Baculovirus

*^a^Peptide sequences used in these fusion proteins were: MBP69–87 (YGSLPQKSQRSQDENPVVH); MBP73–87 (PQKSQRSQDENPVVH); MOG35–55 (MEVGWYRSPFSRVVHLYRNGK); and PLP139–151 (HSLGKWLGHPDKF)*.

*^b^Fusion proteins used to study rat or mouse models of EAE were rat or mouse in origin, respectively. Mouse GMCSF-NAg, both rat and mouse IFNbeta-NAg, and rat IL2-NAg contained a non-native alanine as the second amino acid at the N-terminus to optimize a Kozak translation-initiation site (GCCGCCACCATGG). The signal sequence was the native signal sequence for each cytokine gene product except for rat GMCSF-NAg and NAgIL16 which contained the mouse GM-CSF or the honey bee mellitin (HBM) signal sequence, respectively. Rat MCSF-NAg was comprised of the 33 amino acid signal sequence plus the 220 amino acid N-terminal domain which forms a soluble biologically active homodimer. NAgIL16 was comprised of a N-terminal his-tag, the 69–87 encephalitogenic peptide of MBP, and the rat 118-aa IL-16 cytokine C-terminus*.

*^c^A linker between the cytokine domain and the NAg domain was not needed or not included in the primary protein structure with the exception of the IFNbeta-NAg fusion proteins, where this linker was essential for full expression of IFN-beta activity. For those cytokine-NAg linkers in which the linker was “not needed,” the vaccine was originally expressed with the linker, but subsequent versions that lacked the linker had full activity in assays of cytokine activity, antigenic activity, and tolerance induction*.

Several fusion proteins had both tolerogenic and therapeutic activity (Table 2). When injected subcutaneously in saline before encephalitogenic immunization, GMCSF-NAg, IFNbeta-NAg, NAgIL16, and IL2-NAg prevented or attenuated the subsequent active induction of EAE. When administered after onset of EAE, these vaccines also were effective interventions that blunted the progression of EAE. Because these fusion proteins had both tolerogenic (i.e., preventative) and therapeutic (i.e., inhibition of effector autoimmune responses) activity, these vaccines were referred to as tolerogenic, therapeutic vaccines. The characteristics of these vaccines are summarized in Table 2, and two of these vaccines (GMCSF-NAg and IFNbeta-NAg) are discussed in detail including considerations of relative tolerogenic efficacy, requirement for covalent cytokine-NAg linkage, and differential potency and subset-specificity in targeting of the covalently tethered NAg to APC.

**Table 2:** **Summary of cytokine-NAg vaccines tested in EAE**.

Cytokine-NAg vaccine^a^	Inhibitory efficacy in EAE^a^	Cytokine-NAg linkage needed for:		Antigenic targeting to APC *in vitro*^c^	Citation
		pre-treatment^b^	treatment^b^		
Rat GMCSF-NAg	Yes	Yes	Yes	>1000-fold (myeloid APC)	Blanchfield and Mannie (2010)
Murine GMCSF-NAg	Yes	Yes	Yes	∼10-fold (myeloid APC)	Abbott et al. (2011)
Rat IFNbeta-NAg	Yes	No	No	∼1 to 10-fold (splenic APC)	Mannie et al. (2009b)
Murine IFNbeta-NAg	Yes	Yes	–-^d^	–-^d^	
Rat NAgIL16	Yes	Yes	Yes	∼1 to 10-fold (splenic APC)	Mannie and Abbott (2007)
Rat IL2-NAg	Yes	Yes	Yes	>1000-fold (I-A^+^ T cells)	Mannie et al. (2007)
Rat MCSF-NAg	Yes	Yes	–-	10-100 fold (myeloid APC)	Blanchfield and Mannie (2010)
Rat IL4-NAg	Limited	–-	–-	>(1000-fold (B cell APC)	Mannie et al. (2007)

*^a^Cytokine-NAg vaccines exhibited efficacy in both prevention (vaccine administration before encephalitogenic immunization) and therapeutic (vaccine administration at or after EAE onset) vaccine regimens*.

*^b^Yes: the cytokine-NAg vaccine was tolerogenic but equimolar doses of cytokine and NAg as separate molecules lacked tolerogenic activity. No: the cytokine-NAg vaccine had a tolerogenic efficacy similar to that of equimolar doses of cytokine and NAg*.

*^c^Antigen-targeting: the “fold” enhancement in antigenic potency of a cytokine-NAg vaccine compared to NAg alone in stimulating MHCII-restricted proliferation of a NAg-specific T cell clone in the presence of myeloid APC, non-fractionated splenic APC, blastogenic T cell APC, or purified B cell APC*.

*^d^(–—): Not tested*.

### GMCSF-NAg

Fusion of GM-CSF with the NAg peptides MOG35–55, PLP139–151, or MBP69–87 domains did not quantitatively affect the potency of the GM-CSF cytokine domain (Blanchfield and Mannie, 2010; Abbott et al., 2011). Rather, these GMCSF-NAg fusion proteins were equipotent compared to free GM-CSF in cytokine bioassays. GM-CSF and GMCSF-NAg elicited equipotent proliferation of bone marrow cells in the 1–10 pM range. These findings indicated that GM-CSF is a versatile carrier able to accommodate diverse peptide structures without adverse effect on GM-CSF biological activity.

**Figure 2:**
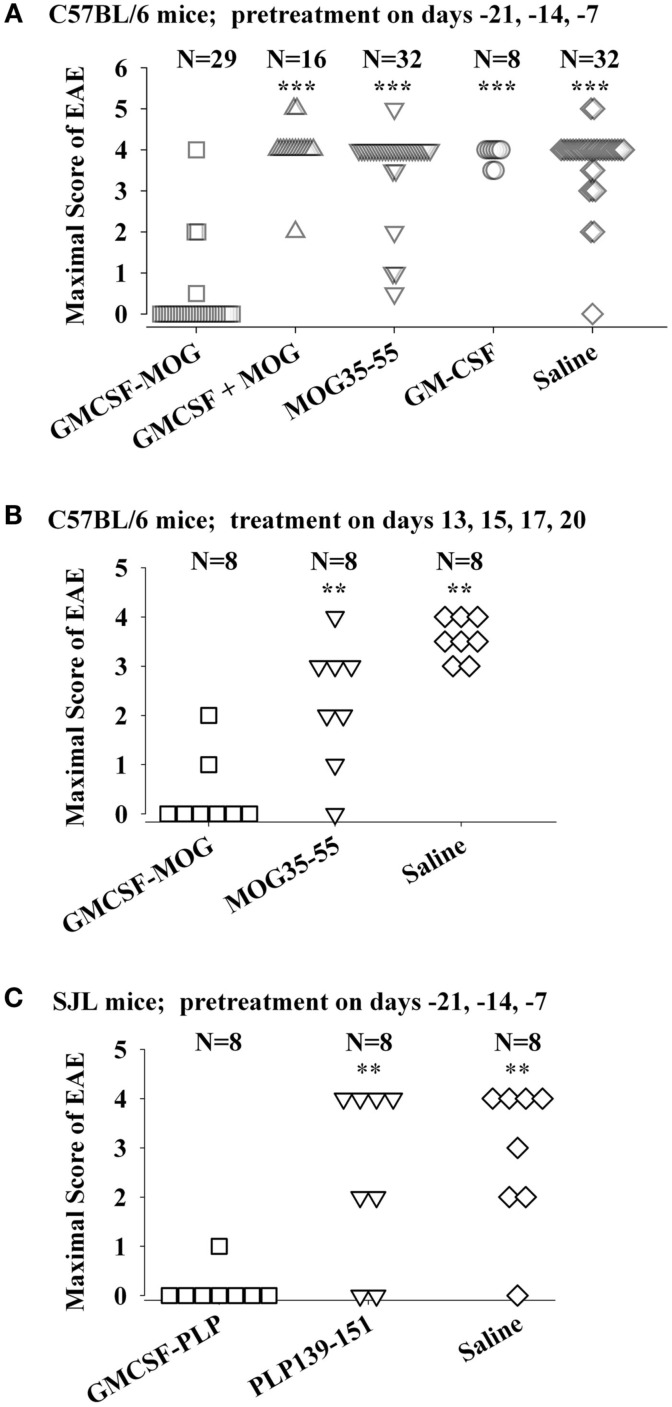
**Mouse cytokine-NAg vaccines were effective tolerogens in the C57BL/6 and SJL mouse models of EAE**. EAE was elicited in C57BL/6 mice by injection of MOG35–55 in CFA together with separate injections (200 ng i.p.) of *Pertussis toxin* on days 0 and 2. EAE was elicited in SJL mice by injection of PLP139–151 in CFA. EAE induction was on day 0 relative to pre-treatment with designated cytokine-NAg fusion protein or control proteins (*x*-axis) on days-21, -14, and -7 **(A,C)** or treatment on days 13, 15, 17, and 20 **(B)**. Vaccines were administered subcutaneously in saline. Maximal disease scores were defined as the most severe disease score exhibited by a mouse throughout the relevant disease course. The mouse clinical EAE scoring scale was: 0, no disease; 1.0, partial or full paralysis of the tail or ataxia but not both; 2.0, flaccid paralysis of the tail and ataxia or impaired righting reflex; 3.0, partial hind-limb paralysis; 4.0, full hind-limb paralysis; 5.0 total hind-limb paralysis with forelimb involvement. *p* Values were calculated by non-parametric ANOVA based on ranked data with a Bonferroni *Post hoc* Test. Data analysis for these experiments was previously reported in (Abbott et al., 2011). Pre-treatment of C57BL/6 mice was portrayed in Tables 1 and 2, pre-treatment of SJL mice was portrayed in Table 3, and treatment of C57BL/6 mice was portrayed in Table 4 of Abbott et al., 2011; ***p ≤ 0.001; **p ≤ 0.01; *p ≤ 0.05; for comparisons of the cytokine-NAg-treated group with the respective control group).

**Figure 3:**
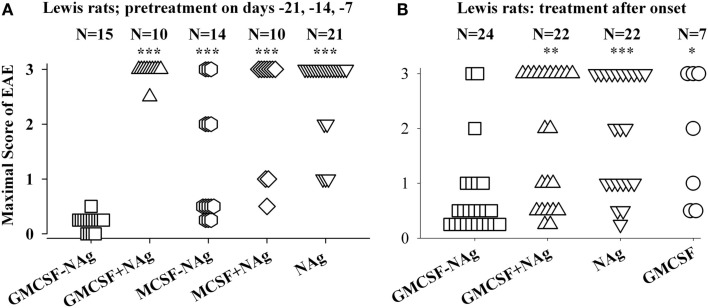
**The rat GMCSF-MBP vaccine was an effective tolerogen in the Lewis rat model of EAE**. EAE was elicited in Lewis rats by injection of NAg in CFA on day 0. Designated cytokine-NAg proteins or control proteins (*x*-axis) were given as pre-treatments on days-21, -14, and -7 **(A)** or alternatively were given as treatments after the onset of paralytic EAE **(B)** as described in (Blanchfield and Mannie, 2010). Vaccines were administered subcutaneously in saline. Maximal disease scores were previously reported in (Blanchfield and Mannie, 2010). The clinical scoring scale for rats was; 0, no disease; 0.25, distal limp tail; 0.5, limp tail; 1.0, ataxia; 2.0, partial hind-limb paralysis; 3.0, full hind-limb paralysis. *p* values were calculated as described in Figure 2. These data were previously portrayed in Tables 1 and 2 respectively of (Blanchfield and Mannie, 2010). (***p ≤ 0.001; **p ≤ 0.01; *p ≤ 0.05 for comparison of the respective treatment groups to that of GMCSF-NAg).

The tolerogenic activity of the GMCSF-NAg vaccines in mouse and rat models of EAE (Blanchfield and Mannie, 2010; Abbott et al., 2011) are portrayed in Figures 2 and 3. These data highlight the efficacy of each treatment group based on maximal disease scores. For example, 86% of C57BL/6 mice pretreated with GMCSF-MOG(35–55) showed no disease whereas mice pretreated with a combination of GM-CSF and MOG35–55 had severe paralytic disease marked by a maximal score of 4.0 or 5.0 (81 and 13% of mice, respectively; Figure 2A). Likewise, over 80% of mice in control pre-treatment groups that received GM-CSF alone, MOG35–55 alone, or saline exhibited severe paralytic disease. Thus, GMCSF-MOG(35–55) had tolerogenic activity because the vaccine effect was remembered by the immune system as an enduring modification of the MOG-specific encephalitogenic response. When administered at the first day of clinical onset, GMCSF-MOG(35–55) was effective as a treatment intervention that halted the subsequent progression of EAE (Figure 2B). The majority of mice treated with GMCSF-MOG did not progress beyond a grade of minimal tail involvement, whereas 100% of mice treated with saline exhibited severe hind-limb paralysis. In this treatment protocol, GMCSF-MOG(35–55) was more effective than “MOG35–55 alone” which in turn was more effective than saline. These data indicate that GMCSF-MOG(35–55) could also intercept the encephalitogenic response during the staging of an attack on CNS myelin. To test the generality of this approach, a murine GMCSF-NAg vaccine was also derived for the SJL EAE model that incorporated the PLP139–151 encephalitogenic peptide as the NAg rather than MOG35–55 (Figure 2C). Subcutaneous injection of GMCSF-PLP(139–151) in saline was shown to prevent EAE in 88% of SJL mice. The remaining GMCSF-PLP pretreated mice had very mild paralysis that was limited to the tail. In contrast, a substantial percentage (≥50%) of mice pretreated with PLP139–151 or saline had severe paralytic disease. These data revealed that GMCSF-NAg vaccines were tolerogenic vaccines in two separate murine models of EAE.

The original study of GMCSF-NAg was performed by use of the Lewis rat model of EAE (Blanchfield and Mannie, 2010). In this study, GM-CSF was compared to M-CSF as a tolerogenic fusion partner by deriving fusion proteins in which the rat GM-CSF or M-CSF was linked to the immunodominant encephalitogenic 69–87 epitope of MBP. The GMCSF-MBP(69–87) vaccine was more tolerogenic than the MCSF-MBP(69–87) fusion protein (Figure 3). All rats that received GMCSF-NAg had no or very mild signs of EAE whereas 42% of rats pretreated with MCSF-NAg exhibited severe paralytic disease (Figure 3A). The finding that GM-CSF was a more effective tolerogenic fusion partner than M-CSF provided suggestive evidence that active induction of myeloid APC was more important than “quiescent maintenance” for induction of tolerance (Fleetwood et al., 2007; Hamilton, 2008). This outcome was expected because GM-CSF facilitates MHCII expression on myeloid APC whereas M-CSF confers viability to the macrophage lineage but does not maintain MHCII expression (Blanchfield and Mannie, 2010). GM-CSF promotes differentiation of MHCII^+^ dendritic cells (DC) whereas M-CSF appears to be a maintenance factor for quiescent or non-inflammatory MHCII^−^ macrophages. Because MHCII expression is critical for presentation of the NAg domain which is in turn critical for induction of tolerance, induction or maintenance of MHCII expression may be a requisite activity of tolerogenic fusion partners.

Covalent linkage of the cytokine and NAg domains was necessary for tolerogenic efficacy. All rats pretreated with GMCSF-MBP(69–87) were protected from severe EAE whereas all rats that received an equimolar mix of GM-CSF and NAg exhibited severe paralysis (Figure 3A). When administered after the onset of clinical signs, GMCSF-MBP(69–87) stopped the progression of EAE by a mechanism that was contingent upon cytokine-NAg linkage (Figure 3B). Physical linkage of GM-CSF and MOG35–55 domains was also necessary for tolerance induction in the C57BL/6 model of EAE (Figure 2). Overall, these data show that GM-CSF is an efficacious tolerogenic fusion partner that facilitates tolerance of covalently attached myelin antigens in two distinct rodent species and in qualitatively different models of EAE. The requirement for covalent linkage of cytokine and NAg domains provides evidence for “antigenic targeting” as a potentially important event in tolerance induction in both pre-treatment and treatment regimens.

The tolerogenic potency of GMCSF-NAg was paradoxical given that GM-CSF is a cytokine closely associated with the induction of EAE in mice (McQualter et al., 2001), and the NAg determinants represent the most potent and dominant encephalitogenic determinants for the respective rodent strains (Mannie et al., 2009a; Miller et al., 2009). Yet, a vaccine generated by the combination of these two pro-encephalitogenic domains comprised a potent tolerogen. Hence, tolerogenic, therapeutic vaccines are not simply a sum of their parts. Rather, synergy of two physically connected domains provides novel activities most likely reflecting unpredicted interactions between APC conditioning and antigenic targeting. GM-CSF has been shown to be critical for EAE induction, but these studies have important caveats in regard to the target tissue localization of pro-disease activity. Indeed, the ability of NAg-specific T cells to produce GM-CSF during activation appears to be a defining, central characteristic underlying T cell-mediated pathogenesis in EAE (Marusic et al., 2002; Ponomarev et al., 2007; Kroenke et al., 2008; Becher and Segal, 2011; Codarri et al., 2011; El-Behi et al., 2011). Localization of GM-CSF to the target tissue, either elaborated by infiltrating T cells or via genetic manipulation, defined an essential aspect of how GM-CSF promotes disease in EAE and other autoimmune diseases (Biondo et al., 2001; Judkowski et al., 2004).

Conversely, administration of GM-CSF inhibits autoimmune disease in mouse models of type I diabetes (Enzler et al., 2003, 2007; Gaudreau et al., 2007, 2010; Meriggioli et al., 2008; Cheatem et al., 2009), myasthenia gravis (Sheng et al., 2006, 2008; Meriggioli et al., 2008), and thyroiditis (Vasu et al., 2003; Gangi et al., 2005; Ganesh et al., 2009, 2011; Bhattacharya et al., 2011). GM-CSF appears to promote differentiation of regulatory DC subsets which in turn facilitate the activity of regulatory T cell subsets that actively inhibit autoimmune disease. Administration of GM-CSF may thereby influence the balance between immunity and tolerance based on factors such as dose, schedule, route, and bio-distribution. Overall, GM-CSF appears important for development and maintenance of regulatory DC-T cell networks. And GMCSF-NAg vaccines appear to target NAg to these networks and thereby cause tolerance rather than immunity to the myelin self-antigen domains.

### IFNbeta-NAg

**Figure 4:**
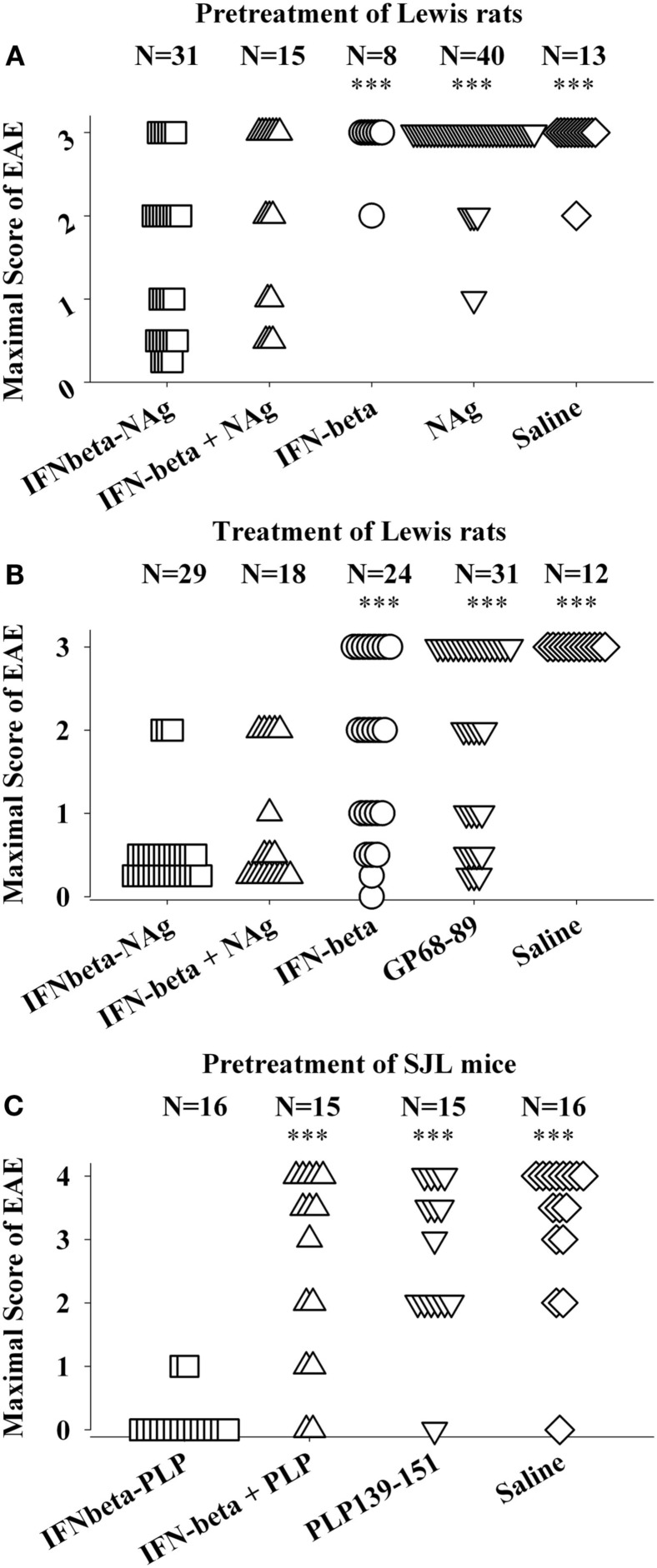
**IFNbeta-NAg vaccines were effective tolerogens in the Lewis rat and SJL models of EAE**. EAE was elicited in Lewis rats or SJL mice by injection of the respective NAg in CFA on day 0. Designated cytokine-NAg proteins or control proteins (*x*-axis) were given as pre-treatments on days-21, -14, and -7 **(A,C)** or alternatively were given as treatments after the onset of paralytic EAE **(B)**. Vaccines were injected subcutaneously in saline. The data analysis for experiments shown in **(A,B)** was previously reported in Tables 2 and 4 (pre-treatment) and Tables 4 and 5 (treatment) in Mannie et al. (2009b). In the pre-treatment protocol **(A)**, Lewis rats that were given the combination of IFNbeta + MBP69–88 as separate injections in adjacent subcutaneous sites had significantly less severe EAE than rats treated with MBP69–88 alone (*p* = 0.001) or saline (*p* = 0.011). In the treatment protocol **(B)**, Lewis rats that were given the combination of IFNbeta + MBP69–88 as separate molecules had significantly less severe EAE than rats treated IFN-beta alone (*p* = 0.001), MBP69–88 (*p* < 0.001), or saline (*p* < 0.001). (***p ≤ 0.001; **p ≤ 0.01; *p ≤ 0.05 for comparison of the respective treatment groups to that of IFNbeta-NAg).

The potent efficacy of IFN-beta as a tolerogenic fusion partner is notable given that this cytokine is used as a first-line therapy for MS (Kieseier, 2011; Killestein and Polman, 2011; Plosker, 2011; Rudick and Goelz, 2011). The rat IFNbeta-MBP(69–87) had strong tolerogenic activity, but in contrast to what was found for GMCSF-NAg and other cytokine-NAg vaccines, an equimolar mix of IFN-beta and NAg was nearly as effective as the IFNbeta-NAg vaccine in both pre-treatment and treatment regimens (Figures 4A,B; Mannie et al., 2009b). The lack of requirement for domain linkage was apparent even when the two domains were injected in separate but adjacent sites. A murine IFNbeta-PLP(139–151) vaccine was derived to test the generality of these findings. The murine IFNbeta-PLP fusion protein also induced tolerance in a pre-treatment protocol in the SJL relapsing-remitting model of EAE (Figure 4C). Unlike rat IFNbeta-NAg however, the murine IFNbeta-NAg required covalently linked cytokine and NAg domains for tolerance induction. In the IFNbeta-PLP(139–151) pre-treatment group, 100% of mice had no or very mild EAE, whereas over 50% of mice pretreated with the combination of murine IFNbeta and PLP139–151 as separate molecules had severe paralytic EAE. Likewise, mice pretreated with PLP139–151 alone or saline also exhibited severe paralytic EAE. Hence, the rat IFNbeta-MBP(69–87) was a notable exception to the rule that these vaccines required covalently linked cytokine and NAg domains. Why these particular rat and murine IFNbeta-NAg had differential requirements for cytokine-NAg linkage is not known. One cannot conclude that these observations represent a species difference given that to date only one IFNbeta-NAg fusion protein vaccine has been tested in each rodent species.

The lack of requirement for covalent linkage in the rat model could be potentially explained by either a direct or indirect binding interaction (perhaps via a third party molecule) by which the peptide became non-covalently associated with IFN-beta. Such an adventitious event would be considered exceptional. An alternative possibility is that the two reagents were sequestered in the same lymphatic drainage where IFN-beta and NAg may synergistically mediate tolerance by a localized or paracrine mechanism. IFN-beta is known to have pronounced anti-proliferative and anti-metabolic activity and thereby may impair clonal expansion and differentiation of NAg-specific effector T cells. Perhaps the potent cytotoxic activity of IFN-beta may inhibit or preempt NAg-specific clonal expansion and differentiation needed to stage an encephalitogenic response and may render the NAg-specific clonotypes anergic or alter their differentiation toward a regulatory phenotype.

Importantly, this study provides evidence that IFN-beta fundamentally alters how the immune system responds to antigen. As reviewed elsewhere (Mannie et al., 2009b), IFN-beta inhibits EAE when administered during the immunization or effector phases of disease. Our studies however indicated that IFN-beta (without NAg) did not exert modulatory activity in EAE when administered on days-21, -14, and -7 before encephalitogenic immunization. Most likely, this pre-treatment protocol had no effect because IFN-beta was presumably cleared from the body before encephalitogenic immunization. The simplest explanation is that IFN-beta will not shape a T cell repertoire or have lasting effects on adaptive immunity unless IFN-beta is present concurrently with an antigen that is driving an immune response. When given with NAg, particularly in the form of a fusion protein, IFN-beta showed properties of a tolerogenic adjuvant. That is, in the presence of NAg, IFN-beta modified immune responsiveness by conferring an enduring immunological tolerance to that NAg. Thus, this study reveals a novel adjuvant activity of IFN-beta that was not previously appreciated as a classical IFN-beta activity. Nonetheless, the action of IFN-beta as a tolerogenic adjuvant is consistent with the efficacy of IFN-beta as a front-line therapeutic in MS. That is, IFN-beta in MS patients may act synergistically with myelin-derived endogenous antigens to promote a lasting tolerogenic activity specific for those antigens. This scenario may provide a rationale for IFN-beta dose escalation during acute relapses of MS, not only to contain the inflammatory demyelination, but also to maximize the bioavailability of IFN-beta during a period when NAg may be released from the CNS into the periphery and would be available to act synergistically with IFN-beta to promote tolerance against those myelin-derived NAg.

### NAgIL16 and IL2-NAg vaccines

NAgIL16 and IL2-NAg were two additional vaccines that had pronounced tolerogenic activity in the Lewis rat model of EAE. Both cytokine fusion partners were originally chosen based on their ability to regulate CD4^+^ T cell biology. IL-16 is a highly conserved, species-cross reactive cytokine (Keane et al., 1998) that may associate with CD4 or other cell surface receptors (Mathy et al., 2000) and has been implicated in chemotaxis of T cells, DC, and other leukocyte subsets (Cruikshank et al., 2000). However, many functions attributed to IL-16 are seen only at high concentrations, and the true biological function of IL-16 remains a mystery. IL-16 is synthesized as a large precursor protein and is cleaved by caspase-3 into a N-terminal portion that is translocated into the nucleus and a C-terminal protein that constitutes the active secreted IL-16 (Zhang et al., 1998, 2001). IL-16 therefore shares characteristics of IL-1-beta, IL-18, and IL-37 in that the cytokine is liberated in the cytoplasm by proteolytic cleavage of a large precursor and then secreted to exert biological functions in the extracellular environment (Takenouchi et al., 2009).

IL-2 was chosen as a fusion partner because IL-2 has a requisite role in the maintenance of self-tolerance based on the ability of IL-2 to promote the differentiation and expansion of regulatory T cell subsets (Malek and Bayer, 2004; Fehervari et al., 2006). For both NAgIL16 and IL2-NAg vaccines, covalent linkage of the cytokine and NAg domains was required for inhibitory efficacy *in vivo* in both pre-treatment and treatment regimens. For these vaccines, administration of equimolar doses of the free cytokine and NAg molecules as a mixture of separate molecules did not cause tolerance. The requirement for covalent linkage of cytokine and NAg domains provides suggestive evidence for a mechanism of “antigenic targeting.”

## Antigen-Targeting Activity of Cytokine-NAg Vaccines

**Figure 5:**
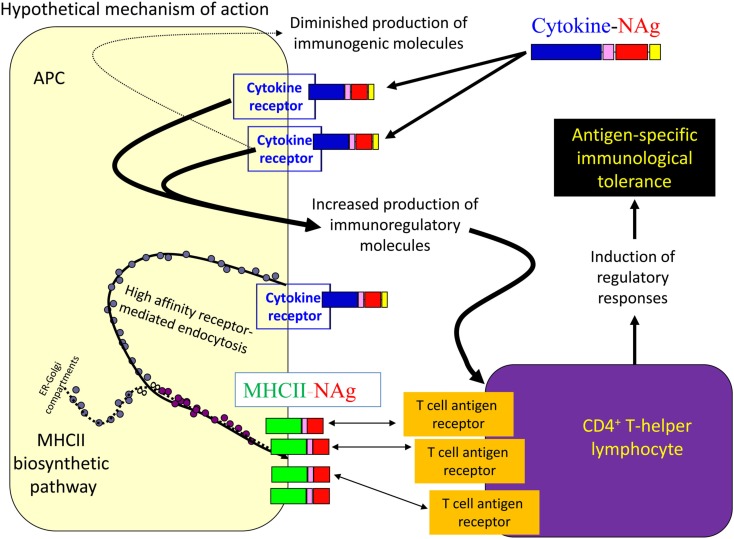
**Mechanism of antigen-targeting by cytokine-NAg vaccines**. The following sequence of events is postulated to mediate the conditioning and targeting activities of cytokine-NAg vaccines. First, the cytokine domain of the vaccine binds to APC subsets that bear the respective cytokine receptor. Engagement of the cytokine receptor on the APC triggers a shift from costimulatory activity to a counter-regulatory or tolerogenic co-inhibitory activity. Engagement of the cytokine receptor also targets the covalently attached NAg domain to the APC. Ingestion of cytokine receptor/vaccine complexes into the endosomal compartment introduces the NAg domain in high concentrations to the MHCII-antigen processing pathway. Processing of the vaccine liberates the NAg domain for loading onto nascent MHCII glycoproteins. By this mechanism, APC that are conditioned by the cytokine domain in turn present high concentrations of NAg domain on MHCII glycoproteins. Tolerogenic presentation of the NAg is postulated to induce regulatory elements and blunt the encephalitogenic immune response.

Several cytokine-NAg vaccines had antigen-targeting activity (Tables 1 and 2; Figure 5). “Antigenic targeting” was based on the observation that rat GMCSF-NAg, IL4-NAg, and IL2-NAg vaccines were ∼1000-fold more potent when compared to NAg alone in assays measuring the MHCII-restricted presentation of NAg by DC, B cells, or blastogenic (rat) T cells, respectively (Mannie et al., 2007; Blanchfield and Mannie, 2010). The following observations provided important insight into mechanisms by which cytokine-NAg vaccines targeted the NAg to APC for enhanced antigen presentation. First, covalent linkage between the cytokine domain and the antigenic domain was needed for potentiated antigen recognition. Addition of cytokine and NAg as separate molecules did not result in enhanced T cell responses. This finding indicated that the cytokine domain did not enhance antigen presentation of NAg by a generic augmentation of antigen processing, MHCII expression, cytokine production, or some other general aspect of APC activity. Second, the antigen-targeting profile of a given cytokine-NAg vaccine was specific for particular subsets of APC. GMCSF-NAg was targeted to myeloid APC and IL4-NAg was targeted to B cells. Antigenic targeting was observed for IL2-NAg when a rat blastogenic T cell clone bearing high surface densities of MHCII and CD25 was used as APC. In these cases, IL2-NAg was ∼1000-fold more active as an antigen than the isolated NAg domain. These data are consistent with the hypothesis that antigen-targeting required a specific interaction between the cytokine domain of the vaccine and the respective cytokine receptor on the APC surface. Third, antigen-targeting was blocked by the addition of free cytokine. For example, potentiated antigenic recognition of GMCSF-NAg was blocked by GM-CSF but not by M-CSF whereas potentiated antigenic recognition of MCSF-NAg was blocked by M-CSF but not by GM-CSF (Blanchfield and Mannie, 2010). Enhanced antigenic recognition of IL4-NAg was blocked by IL-4 or an anti-IL-4 mAb. Likewise, the enhanced antigenic recognition of IL2-NAg was blocked by IL-2 (Mannie et al., 2007). These findings indicate that the high affinity docking of the cytokine domain to cytokine receptors on APC was the key event for potentiated antigen recognition. Lastly, the T cell proliferative response to the cytokine-NAg vaccine was blocked by MHCII-specific antibodies, indicating that the enhanced T cell proliferative response was due to MHCII-restricted recognition of NAg rather than an independent mitogenic activity of the cytokine domain. These data support the concept that the cytokine domain of a cytokine-NAg vaccine binds the respective receptors on select APC subsets to facilitate the uptake of the NAg domain into the MHCII-antigen processing pathway, where the NAg is liberated and loaded onto nascent MHCII glycoproteins (Figure 5). The cytokine receptor-mediated, high affinity, high-capacity uptake of these vaccines by APC is postulated to account for the enhanced presentation of NAg to NAg-specific T cells.

Two observations revealed potential associations between antigenic targeting and tolerance induction. First and most importantly, most cytokine-NAg vaccines required covalently linked cytokine-NAg domains for tolerance induction (Table 2). GMCSF-NAg (both rat and mouse versions), mouse IFNbeta-NAg, MCSF-NAg, NAgIL16, and IL2-NAg required covalently linked cytokine-NAg domains for tolerance induction, although rat IFNbeta-NAg did not, and as discussed above, the reason for this exception is currently unknown. Second, most vaccines showed some degree of antigenic potentiation compared to NAg alone in assays measuring MHCII-restricted stimulation of NAg-specific T cell clones. However, the quantitative magnitude of antigenic targeting varied substantially among different cytokine-NAg vaccines. Rat GMCSF-NAg and IL4-NAg exhibited profound antigenic targeting in the presence of irradiated splenic APC, but rat GMCSF-NAg was a potent tolerogen whereas IL4-NAg lacked tolerogenic efficacy. Given that GMCSF-NAg targeted NAg to myeloid APC whereas IL4-NAg targeted NAg to B cell APC, the implication is that the APC subset targeted by the vaccine may be a critical variable in the efficiency of tolerance induction. These issues however may be complicated and are currently unresolved. For example, the ability of IL-4 to confer an immunogenic phenotype to DC and target NAg to those DC may counteract any tolerogenic effects associated with the interaction of IL4-NAg with B cells.

The antigenic potentiation associated with mouse GMCSF-NAg, rat NAgIL16, and IFNbeta-NAg (either mouse or rat) was 10-fold or less in the presence of irradiated splenic APC, but these fusion proteins were effective tolerogens. Thus, antigenic targeting may be an important component of the tolerogenic mechanism, at least for certain vaccines, but cannot be considered a sole factor determining the extent of tolerance induction. Thus, the cytokine domain, in addition to facilitating antigenic targeting to certain APC subsets, may also condition the APC to favor tolerogenic presentation. Overall, the following inter-related concepts may important for understanding the tolerogenic activity of cytokine-NAg fusion proteins; (a) the quantitative degree of antigenic targeting, (b) the APC subset targeted by a given vaccine, and (c) cytokine-mediated conditioning of that APC subset. Combinations of these qualities, and perhaps others not yet realized, may be central to understanding the mode of action. Given that GM-CSF, IFN-beta, IL-16, and IL-2 have unique and highly diverse activities, one cannot assume a common tolerogenic mechanism for these diverse vaccine products.

## Attributes of NAg-Specific Cytokine-NAg Vaccines

### The cytokine domain as an amplifier of a tolerogenic antigen domain

All tested cytokine-NAg vaccines had some degree of inhibitory activity *in vivo*. Notably, none of the cytokine-NAg fusion proteins augmented EAE (Mannie and Abbott, 2007; Mannie et al., 2007, 2009b; Blanchfield and Mannie, 2010; Abbott et al., 2011). In studies by other groups, many of the same cytokine domains, particularly GM-CSF, were coupled to foreign proteins and were found to augment immunity to those foreign proteins (Wortham et al., 1998; Rodriguez et al., 1999; Tso et al., 2001; Wang et al., 2009; Zhai et al., 2009; van Montfort et al., 2011). At the outset of this project, the anticipation was that the cytokine domain would be more important than the antigenic domain for determining the balance of tolerance versus immunity. However, the opposite possibility should be considered. The cytokine domain, in part, may be an amplifier of the intrinsic immunogenic or tolerogenic activity of the covalently coupled antigenic domain. This may be why GM-CSF has been used as a fusion partner to augment immunity or tolerance depending on the foreign or “self” origins of the antigenic domain. The possibility is that cytokines target the antigenic domain to selected APC subsets for enhanced presentation, and the relative balance of conventional and regulatory T cells that recognize those epitopes determines the outcomes of immunity or tolerance. Foreign and self-antigen domains would be preferentially recognized by conventional and regulatory subsets respectively, and these initial interactions may dictate the type and scope of the ensuing immune response. The cytokine domain may also affect the balance of immunity and tolerance through mechanisms of APC conditioning, and these APC conditioning events may be reinforced by the relative balance of T cell subsets that initiate and subsequently dominate the response. In turn, early T cell biasing events may polarize additional APC and by such a feed-forward mechanism determine the immunogenic or regulatory activities of the overall immune response. Thus, vaccine activity cannot be predicted simply based on the isolated activity of the cytokine domain. Rather, the activities of the cytokine, the antigenic domain, and their interaction, particularly in regard to mechanisms of APC conditioning, antigenic targeting, and early biasing of T cell subsets may be the key considerations of antigen-specific tolerogenic efficacy.

Because a substantial percentage of all cytokine-NAg vaccines tested in the rat model had significant tolerogenic efficacy, the extrapolation is that many other highly efficacious cytokine fusion partners may exist in addition to the ones tested so far in EAE. Overall, the field of cytokine-NAg vaccines may have substantial promise for a diversity of chronic inflammatory conditions based on drug discovery of an expanded set of suitable cytokine fusion partners. The concept is that many other cytokines may be useful as fusion partners for the induction of tolerance and that the findings to date simply represent the tip of the iceberg in broaching the use of cytokine-antigen fusion proteins for the induction of tolerance.

### The cytokine domain

The following characteristics of the cytokine domain may favor induction of tolerance. The cytokine domain should mediate efficient antigen-targeting to an APC subset associated with the induction of tolerance. The caveat is that no APC subset is solely dedicated to tolerance but some may facilitate tolerance more efficiently than others. The cytokine domain should be compatible with the induction and maintenance of MHCII expression. Conversely, cytokines that down-regulate MHCII glycoproteins may be suppressive but not tolerogenic and may not represent desirable fusion partners. Cytokines that are highly stable and soluble will facilitate expression, purity, and yield of the protein. The cytokine fusion partner should have a C-terminus or N-terminus apart from the active site so that extension of one terminus with an antigenic peptide will not impair cytokine activity. For example, an IL16-NAg fusion protein (NAg at the C-terminus) was less effective as a NAg than an alternative version (NAgIL16) that placed the NAg domain at the N-terminus.

### The NAg domain

The following characteristics of the NAg domain may favor tolerance. The NAg domain should be a dominant encephalitogenic epitope of myelin, or more broadly, a major autoimmune epitope. The concept of antigen-specific tolerance in autoimmune disease is contingent upon “hitting the nail on the head.” One would surmise that targeting minor epitopes would have little effect on the autoimmune disease unless such determinants subsequently became important in perpetuating chronic disease. An inter-related consideration is that the NAg should be “self” to facilitate recognition by regulatory T cells which favor self-recognition. The NAg domain should be soluble to minimize protein aggregation and facilitate expression, purification, and yield of the recombinant protein. This requirement of solubility for this vaccine approach may preclude use of peptide epitopes buried in transmembrane or hydrophobic domains.

### Linkage of the cytokine-NAg domains

Most cytokine-NAg vaccines did not require linkers between the cytokine and NAg domains (Table 1). GMCSF-NAg, MCSF-NAg, NAgIL16, IL2-NAg, or IL4-NAg did not require an intervening linker for antigen-targeting activity *in vitro* or for tolerance induction *in vivo*. The notable exception was IFNbeta-NAg in which the enterokinase (EK) linker was needed to preserve full IFN-beta activity (Mannie et al., 2009b). Direct linkage of IFN-beta to the MBP69–87 peptide or conversely, placement of the MBP peptide at the N-terminus resulted in a vaccine characterized by substantial losses of IFN-beta potency. Two considerations should guide research on optimal linker usage in future cytokine-NAg vaccines. First, one would want to avoid extraneous foreign linker sequences, unless necessary for full cytokine domain activity, because such linkers may be immunogenic and elicit neutralizing antibody against the vaccine. Second, the use of linkers with protease-recognition sites may facilitate cleavage and release of the NAg domain in the MHCII-antigen processing pathway and thereby facilitate antigen-targeting and tolerogenic activity of these vaccines.

### Route of administration

Administration of antigen in the absence of adjuvants or co-stimulation has long been associated with the induction of tolerance in many experimental systems including EAE. Myelin-derived peptides and proteins have been administered by nasal, oral, or other mucosal routes, and these routes of NAg administration often result in myelin-specific tolerance capable of blocking EAE (Shi et al., 1998; Miyamoto et al., 2005; Song et al., 2006; Weiner et al., 2011). Other tolerance induction strategies appear contingent upon intravenous delivery of antigen. For example, antigen-coupled leukocytes were highly tolerogenic when delivered intravenously but were immunogenic and promoted EAE when injected by a subcutaneous route (Getts et al., 2011). Other routes of administration also favor tolerance induction. For example, application of myelin antigen on epicutaneous patches elicited active regulatory mechanisms that inhibited EAE (Bynoe et al., 2003). Intrathymic injection of myelin antigen augmented induction of tolerance and inhibited EAE (Khoury et al., 1993; Goss et al., 1994). These data indicate that soluble protein/peptide antigens may induce tolerance by mechanisms contingent on route and may lack effectiveness or tolerogenic potency when delivered via a subcutaneous route.

Subcutaneous administration can have important advantages. Avoidance of the intravenous route should minimize the prospect of adverse anaphylactic reactivity. Administration via a subcutaneous route, as opposed to mucosal or cutaneous application, should aid accuracy in dosing. Because cytokine-NAg vaccines are intended to be given as a limited number of applications, the ease-of-use benefits of mucosal application are minimal. Administration in saline without adjuvants should minimize unintended immunogenic responses that favor the induction of autoimmune disease. To date, subcutaneous injection of cytokine-NAg vaccines has not elicited any sign of local reactivity at the injection site. The finding that cytokine-NAg vaccines can induce tolerance by a limited number of low-dose injections precludes the need for chronic administration and therefore minimizes the likelihood of eliciting neutralizing antibodies against the vaccine. Thus, a primary advantage is that these vaccines were effective tolerogens when given in saline as a limited number of administrations by the subcutaneous route.

### The issue of neutralizing anti-vaccine antibodies

The issue of neutralizing anti-vaccine antibodies was explored for GMCSF-NAg, because the generation of anti-GM-CSF antibodies, unlike anti-IFN-beta antibodies, might inhibit EAE, and confound data interpretation. Several considerations inherent in the experimental approach minimized the possible production of anti-vaccine antibodies, including use of syngeneic “self” cytokine domains, lack of adjuvant, low-dose administration, and a limited number of “boosts.” Nonetheless, rat GMCSF-NAg was expressed from baculovirus-infected insect cultures, whereas the mouse GMCSF-NAg and all IFNbeta-NAg preparations derived from mammalian cell culture (human embryonic kidney cells). Possibly, insect specific-glycosylation of rat GMCSF-NAg may be sufficiently foreign to evoke antibody against this recombinant protein, and possibly, by epitope spread, may give rise to antibodies that could neutralize endogenous GM-CSF to inhibit EAE. However, this was not the case. Pre-treatment with GMCSF-NAg did not result in detectable antibody against either the GM-CSF or NAg domain or any aspect of the vaccine protein (including the histidine-tag). Indeed, subcutaneous treatment of GMCSF-NAg in an alum adjuvant or in combination with either pertussis toxin or TNF-alpha did not elicit detectable antibody against the vaccine protein. Intravenous administration of GMCSF-NAg also did not elicit anti-vaccine antibody. Even high doses (five injections of 8.0 nmol GMCSF-NAg) did not elicit detectable antibody. Immunization of rats with NAg/CFA elicited antibody against the NAg peptide that cross-reacted against the NAg domain of GMCSF-NAg, but this antibody did not interfere with the therapeutic activity of GMCSF-NAg in EAE. The lack of anti-GM-CSF antibody production was not surprising, because GM-CSF was a self protein, and the GMCSF domain lacked the necessary adjuvant activity for immunogenic responses or EAE induction. Thus, our data to this point indicated that anti-GMCSF antibody played no substantial role in the inhibitory activity of GMCSF-NAg.

### Vaccine-mediated inhibitory activity in pro-inflammatory environments

**Figure 6:**
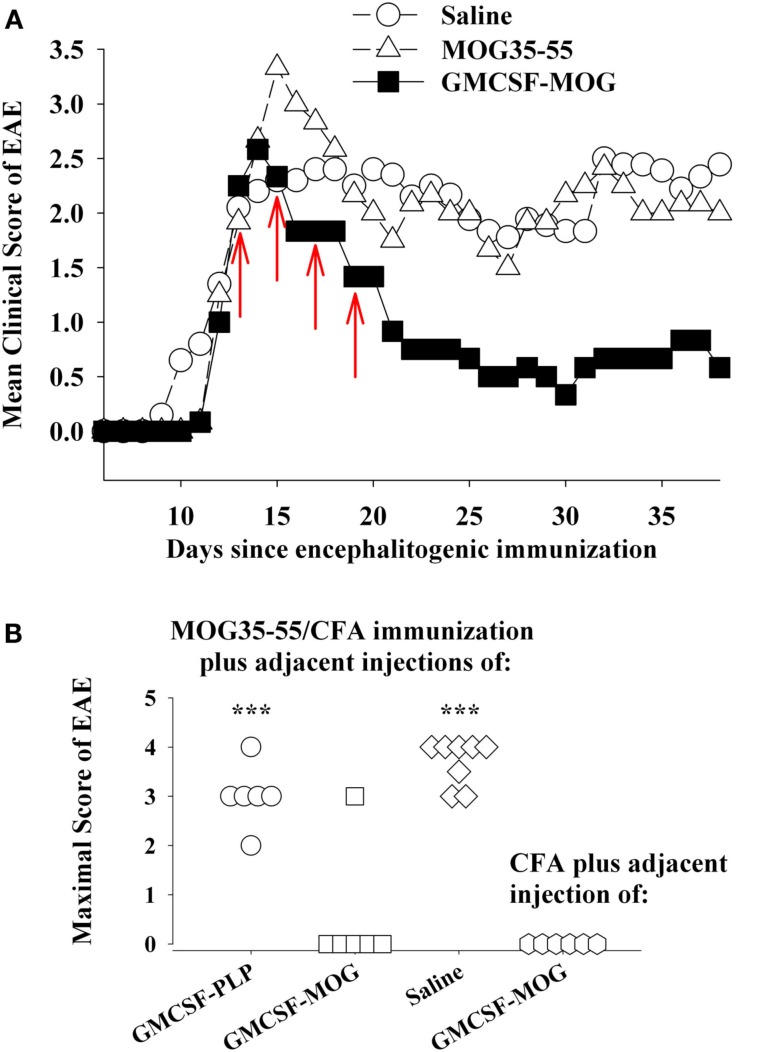
**GMCSF-MOG was an effective therapeutic in pro-inflammatory environments**. **(A)** Mice were immunized on day 0 with 200 μg MOG35–55 in CFA and were also given Pertussis toxin (200 ng i.p.) on days 0 and 2. When the majority of mice began showing paralytic EAE, mice were matched for clinical signs of EAE and were injected with the synthetic peptide MOG35–55, GMCSF-MOG (2 nmol subcutaneously in saline), or saline on days 12, 14, 16, and 18 (red arrows). Mean maximal disease scores of mice treated with GMCSF-MOG significantly differed from those treated with saline (*p* = 0.015; non-parametric ANOVA based on ranked scores). **(B)** On day 0, mice were given an injection of MOG35–55 in CFA to elicit EAE along with an adjacent injection (∼1 mm apart) of 2 nmol GMCSF-PLP, GMCSF-MOG (in saline), or saline. A separate group of mice were injected with a saline/CFA emulsion and an adjacent injection of 2 nmol GMCSF-MOG. All four groups also received Pertussis toxin (200 ng i.p.) on days 0 and 2. Maximal EAE scores of mice given adjacent injections of “MOG35–55/CFA and GMCSF-MOG (second column)” or “saline/CFA and GMCSF-MOG (fourth column)” differed from those for mice injected with MOG35–55/CFA and either GMCSF-PLP (first column) or saline (third column; *p* ≤ 0.001).

GMCSF-NAg was shown to inhibit the effector phase of EAE (Figure 2B). In this experiment, treatment was initiated when the first mice began to show initial signs of EAE. At this point though, a majority of mice remained clinically normal but disease onset was imminent because, in the next 2 days, seven of eight mice developed EAE in the saline-treated group. Treatment with GMCSF-MOG halted progression of EAE in all mice including those showing early signs of EAE. This experiment did not however address whether GMCSF-MOG, delivered subcutaneously in the flank, could down-regulate effector cells already entrenched within the CNS to inhibit established EAE. The experiment shown in Figure 6A was designed to address this question. Treatment with GMCSF-MOG was initiated in a group of mice showing 100% disease incidence and an average severity score of greater than 2. Here again, treatment with GMCSF-MOG reversed the course of EAE and blocked disease progression over the next 3 weeks, even after cessation of treatment. This observation provided evidence that GMCSF-MOG has lasting inhibitory activity despite ongoing inflammation in the CNS.

A common assumption is that quiescent, non-inflammatory environments enable tolerogenic responses whereas pro-inflammatory environments foster immunogenic responses. Based on this assumption, a cytokine-NAg vaccine should exert tolerogenic activity in a quiescent, non-inflamed site but would exhibit immunogenic activity in an inflamed locale. The credence of this assumption however may be lacking, given that appreciable frequencies of potentially pathogenic, self-reactive T cells circulate in most individuals (Elong Ngono et al., 2012), and pro-inflammatory environments are common in most individuals due to infectious disease, injury, or vaccination. Yet prevalence of autoimmune disease is relatively low.

Thus, an important question is whether cytokine-NAg vaccines require non-inflammatory environments for induction of tolerance. For example, if GMCSF-MOG was given to a truly inflamed tissue, would GMCSF-MOG potentiate EAE rather than tolerance? One possible explanation for the ability of GMCSF-MOG to inhibit the effector phase of EAE is that peripheral injection of GMCSF-MOG positions the vaccine within sterile non-inflammatory environments of isolated peripheral lymphoid tissues, separate from the lymphatic drainage associated of the encephalitogenic CFA emulsion and apart from the inflamed CNS. To more directly address this question, GMSCF-MOG (in saline) was given as a single injection immediately adjacent to an active immunization with MOG35–55 in CFA. These mice were also injected with Pertussis toxin to elicit EAE. The adjacent injection of GMCSF-MOG, but not GMCSF-PLP or saline, inhibited the MOG35–55/CFA sensitization as shown by a lack of EAE in five of six mice (second column, Figure 6B). The MOG domain of the GMCSF-MOG vaccine was critical for inhibition, because GMCSF-PLP had no effect on MOG35–55/CFA sensitization (first column). Thus, the inhibitory mechanism was MOG-dependent and antigen-specific. The GMCSF-MOG vaccine was inhibitory within a lymphatic drainage conditioned by CFA and in the midst of CFA-conditioned inflammatory responses. These data indicate that GMCSF-MOG retained regulatory activity within the staging sites of an encephalitogenic response. Overall, these data do not readily fit the concept that a generic pro/anti-inflammatory balance determines the outcome of a tolerogenic vaccination.

A related question was whether GMCSF-MOG had pro-encephalitogenic activity when given in combination with a saline/CFA emulsion when no antigen was actually incorporated into the CFA emulsion (fourth column, Figure 6B). In this case, the saline/CFA emulsion and the GMCSF-MOG would reach the same draining lymphatics. One possibility is that the pro-inflammatory influence of the CFA emulsion would impose a pro-inflammatory outcome on T cells that recognized the MOG35–55 peptide derived from the GMCSF-MOG vaccine, given that the CFA antigens and GMCSF-MOG would likely be processed by many of the same DC. If the CFA emulsion and the GMCSF-MOG vaccine affect the same subset of DC, then one might predict that GMCSF-MOG would cause EAE in this experiment. However, EAE was not detected in this group. Overall, arguments based on a generic pro/anti-inflammatory balance are not sufficient to account for activity of these vaccines. Rather, GMCSF-MOG has strong inhibitory activity that can directly inhibit encephalitogenic responses, even in the midst of a pro-inflammatory environment.

In contrast to GMCSF-MOG, it is notable that GMCSF-PLP did not affect EAE (compare first and second columns, Figure 6B) when given as side-by-side injections with MOG35–55/CFA. The observation underscores the antigen specificity of cytokine-NAg vaccines. Importantly, the GM-CSF domain did not augment EAE when given in the same peripheral drainage site as the MOG35–55/CFA emulsion. The same result was revealed by experiments comparing GMCSF-NAg to GM-CSF (Figures 2A and 3B). Peripheral administration of low-dose GM-CSF did not augment an encephalitogenic response. According to these experiments, administration of GM-CSF in limited doses at peripheral sites in wildtype mice does not augment EAE. These findings show that peripheral administration of GM-CSF does not feed the pro-encephalitogenic CNS actions of GM-CSF. Indeed, peripheral administration of exogenous GM-CSF inhibits autoimmune disease by facilitating differentiation of homeostatic DC-Treg networks (Enzler et al., 2003, 2007; Vasu et al., 2003; Gangi et al., 2005; Sheng et al., 2006, 2008; Gaudreau et al., 2007, 2010; Meriggioli et al., 2008; Cheatem et al., 2009; Ganesh et al., 2009, 2011; Bhattacharya et al., 2011).

These observations underlie a paradox. The GM-CSF domain of GMCSF-MOG is an optimal fusion partner for inhibition of EAE whereas GM-CSF, produced by re-activated CNS-infiltrating T cells, is needed for effector responses of EAE in the CNS (Marusic et al., 2002; Ponomarev et al., 2007; Kroenke et al., 2008; Becher and Segal, 2011; Codarri et al., 2011; El-Behi et al., 2011). One possible explanation for this paradox is that the GMCSF-MOG vaccine acts in peripheral lymphoid tissues to inhibit the generation of MOG-specific T cells needed for induction of EAE, thereby accounting the pre-treatment efficacy of the vaccine. GMCSF-MOG may also impair generation of MOG-specific T cells needed for the maintenance or replenishment of CNS-resident T cells during chronic EAE, thereby explaining the treatment efficacy of the vaccine. In both cases, GMCSF-MOG may be acting peripherally to mitigate disease of the CNS. Conversely, the elaboration of the GM-CSF cytokine in the CNS target tissue may be needed for phagocytic destruction of myelin during the effector phase of EAE. Inhibitory and encephalitogenic activities of GM-CSF are not contradictory, but rather may merely reflect different aspects of GM-CSF in different places at different phases of the immune response. That is, GM-CSF in peripheral sites including secondary lymphoid organs may be homeostatic whereas GM-CSF acting centrally in the CNS may be pathogenic.

### Role of myeloid APC and DC in antigen-specific tolerance

The use of antigen to induce antigen-specific immunological tolerance has been extensively studied over many decades and represents the basis for development of directed therapies for autoimmune disease and other allergic or inflammatory disorders. For autoimmune disease, the simplest reductionist approach is to use a cocktail of peptides representing the major autoantigens that are suspected of driving the autoimmune process (Wraith, 2009). Many variations on this theme exist, including incorporation of DNA sequences encoding major autoantigens into DNA based vaccines (Steinman, 2010). Autoantigen peptides have been coupled to leukocyte cell surfaces, and leukocyte-antigen preparations have been shown to exert tolerogenic activity that prevents autoimmunity and hypersensitivity in rodent models of disease (Turley and Miller, 2010). For peptide and DNA vaccines, DC or related myeloid-derived APC appear to be pivotal APC for induction of tolerance. For cell-based vaccines, myelin peptides are coupled to leukocytes by means of a fixative which causes apoptosis, and tolerance appears dependent upon uptake of apoptotic donor APC by recipient myeloid-derived APC followed by reprocessing and presentation of the myelin antigens by splenic macrophages. These antigen-based approaches have now progressed into clinical trials to assess safety and efficacy.

Our experiments consistently show that cytokine-NAg vaccines are qualitatively superior to the free antigenic peptide for induction of tolerance and inhibition of autoimmunity (Figures 2–4 and 6A). Free antigenic peptides in some cases had some inhibitory activity, but based on the delivery regimens used in our studies, auto-antigenic peptides were useful primarily as “negative” controls. Likewise, comparison of a multivalent concatemer comprising of a linear array of MS-relevant epitopes was substantially more tolerogenic than the individual peptides (Kaushansky et al., 2011). The enhanced efficacy of the multi-epitope protein most likely reflected, in part, protection from proteolysis, enhanced uptake, and perhaps altered processing by APC. These factors likely facilitated a greater exposure of the vaccine to the immune system and thereby promoted more efficient homeostatic regulation of the relevant autoreactive T cell clones.

Several approaches have been devised to develop tolerogenic fusion proteins that incorporate autoantigen into larger carrier proteins. These fusion proteins are designed to target autoantigen into the antigen processing pathways of specialized APC to enhance MHCII-restricted antigen presentation to thereby optimize tolerogenic potency and efficacy. For example, myelin peptides have been incorporated as the CDR3 region of the immunoglobulin heavy chain (Legge et al., 2001; Divekar et al., 2011). These Ig-antigen fusion proteins, when appropriately aggregated and multimerized, interact with low affinity, counter-regulatory Fc-gamma receptors (i.e., CD32) to target antigen for enhanced presentation by a mechanism that results in tolerance to those myelin antigens. Likewise, B lymphocytes were engineered to express immunoglobulin-autoantigen fusion proteins. When introduced into mice, these engineered B cells were able to suppress a number of autoimmune diseases including EAE by an antigen-specific mechanism associated with the presentation of endogenously processed peptides on MHCII glycoproteins in the context of B7.2-mediated co-stimulation (Melo et al., 2002; Xu and Scott, 2004; Zhang et al., 2010). The role of B cells as potentially tolerogenic APC was supported by the observation that specific targeting of encephalitogenic myelin peptide to B cells *in vivo* inhibited the subsequent induction of EAE (Day et al., 1992; Saoudi et al., 1995). In these studies, an encephalitogenic MBP peptide was covalently coupled to anti-IgD antibodies or F(ab’)_2_ anti-IgD. These antigen-anti-IgD proteins were therefore designed to bind to most B cells without interference from secreted antibody and without diversion to Fc receptors. These proteins were used to show a relationship between B cell-targeted antigen presentation and tolerance induction. Overall, these experimental systems reinforce the concept that myelin-derived “self” antigens can be targeted to specialized APC subsets to efficiently introduce those antigens into the MHCII-antigen processing pathway to reinforce self-tolerance and blunt autoimmunity.

In addition to B cells, myelin antigens targeted to DC also suppress EAE. Myelin peptide antigens have been incorporated into the C-terminus of immunoglobulin molecules specific for cell surface molecules on DC such as DEC-205 (Hawiger et al., 2004; Stern et al., 2010). The incorporation of either MOG35–55 or the PLP139–151 peptide at the C-terminus of an anti-DEC-205 antibody resulted in the targeted presentation of those myelin peptides by DC and induction of tolerance and prevention of EAE. Pre-treatment of C57BL/6 mice with the anti-DEC-205-MOG fusion protein prevented the subsequent induction of EAE and induced unresponsiveness in MOG-specific T cells by a mechanism associated with enhanced expression of CD5 on anergic T cells. Pre-treatment with the anti-DEC-205-PLP fusion protein attenuated the subsequent course of EAE in association with anergy of PLP effector cells and emergence of regulatory CD4 + T cells. Together with our studies of GMCSF-NAg in rat and mouse models of EAE, these data provide suggestive evidence that targeting “self” myelin peptides to DC *in vivo* results in antigen-specific tolerance coupled with inhibition of EAE. GMCSF-NAg is of interest because the vaccine is relatively small in size and has robust activity when administered after disease onset. The small size facilitates protein expression and may be advantageous in terms of tissue penetrance and bioavailability.

It is interesting to note that myelin antigens targeted to major APC subsets such as B cells and DC resulted in tolerance able to suppress EAE in various models. Nonetheless, B cells and DC are critical APC cell types that underlie immunogenic responses against infectious agents. The major APC subsets of the immune system therefore do not appear dedicated to either tolerogenic or immunogenic outcomes. Rather, the functional outcome is a complex interplay among many poorly understood factors including the nature of the antigen (self versus non-self) and the carrier and their interaction with the local and systemic environment. Despite this complexity, it is becoming evident that new classes of experimental vaccines can be designed to target major APC subsets to efficiently induce antigen-specific tolerance and thereby control autoimmune and other inflammatory diseases.

## Conclusion

Cytokine-NAg vaccines represent a novel approach for the induction of antigen-specific tolerance and may be useful as a therapeutic approach for MS. This approach has numerous advantages as reviewed in Table 3. This vaccine approach may also be applied to develop therapeutics for other autoimmune diseases. This approach to date has only been superficially explored and requires future research to understand the underlying tolerogenic mechanisms, to test an expanded number of tolerogenic cytokine fusion partners and myelin epitopes, and translate the concept to the derivation of optimal human cytokine-NAg vaccines for treatment of MS.

**Table 3:** **Summary of cytokine-NAg vaccines**.

Attribute	Utility
**ATTRIBUTES AND UTILITIES OF CYTOKINE-NAG VACCINES**	
Antigen-specific induction of tolerance	Alleviate need for broad-spectrum immunosuppressive drugs
Long-lasting protection	Alleviate need for chronic administration
Potency and efficacy in pre-treatment regimens	Vaccines inhibit staging of disease
Potency and efficacy in treatment regimens	Vaccines block the effector phase of disease
Effective subcutaneous administration in saline	Route of vaccination safe and practical
Three injections sufficient to inhibit EAE	Short-term administration minimizes induction of neutralizing antibodies
No adverse reactions at the injection site	No recall inflammatory response or Arthus reaction
Small stable proteins, robust expression systems	Readily expressed and purified, small proteins have advantageous tissue penetrance and bio-distribution
IFN-beta re-purposed as a cytokine domain for myelin-specific vaccines	Extensive clinical experience with IFN-beta as a front-line therapeutic for MS
GM-CSF re-purposed as a cytokine domain for myelin-specific vaccines	Extensive clinical experience with GM-CSF for bone marrow engraftment and stem cell mobilization

## Conflict of Interest Statement

The authors declare that the research was conducted in the absence of any commercial or financial relationships that could be construed as a potential conflict of interest.

## Author Note

Animal care and use was performed in accordance with approved animal use protocols and guidelines of the East Carolina University Institutional Animal Care and Use Committee.

## Abbreviations

DC, dendritic cell(s); EAE, experimental autoimmune encephalomyelitis; GM-CSF, granulocyte-macrophage colony-stimulating factor; IFN, interferon; MBP, myelin basic protein; MHCII, major histocompatibility complex class II; MOG, myelin oligodendrocyte glycoprotein; MS, multiple sclerosis; NAg, neuroantigen(s); PLP, proteolipid protein.
